# PODXL, negatively regulated by KLF4, promotes the EMT and metastasis and serves as a novel prognostic indicator of gastric cancer

**DOI:** 10.1007/s10120-018-0833-y

**Published:** 2018-05-10

**Authors:** Jing Zhang, Zhonglin Zhu, Huijing Wu, Zhilong Yu, Zeyin Rong, Zai Luo, Yiwei Xu, Kejian Huang, Zhengjun Qiu, Chen Huang

**Affiliations:** 1 Department of General Surgery, Shanghai General Hospital, School of Medicine, Shanghai Jiao Tong University, Shanghai, 200080 China; 2 Department of General Surgery, Tongji Hospital, Tongji University School of Medicine, Shanghai, 200065 China; 3 Department of Medical Affairs, Shanghai General Hospital, Shanghai Jiao Tong University, Shanghai, 200080 China; 4 Department of Anesthesiology, Wan Nan Medical College, Wuhu, 241002 China

**Keywords:** KLF4, PODXL, EMT, Metastasis, GC

## Abstract

**Background:**

Podocalyxin-like 1 (PODXL) was reported to be closely associated with the development of various cancers, yet its functional roles and molecular mechanisms remain vague. The aim of our study was to investigate the clinical significance, biological function and molecular mechanism of PODXL in gastric cancer (GC).

**Methods:**

The level of PODXL in GC tissues was detected applying GC tissues microarray, fresh GC tissues and public database (Oncomine). The invasion, metastasis and tumorigenesis role of PODXL were performed in vitro and in vivo. The correlations between KLF4 and PODXL was determined in GC tissues microarray and fresh GC tissues, and the molecular regulatory mechanism between KLF4 and PODXL was explored in vitro.

**Results:**

The high level of PODXL was detected in GC tissues with advanced T stage, lymph node metastasis, Union for International Cancer Control stage and poor differentiation. And Cox proportional hazards model revealed that PODXL can serve as an independent prognostic indicator for disease-free survival and overall survival of GC patients. Moreover, downregulation of PODXL could inhibit EMT and reduce invasion and metastasis in vitro as well as tumorigenesis in vivo. Additionally, our findings showed that PODXL may be a significant downstream target of KLF4.

**Conclusions:**

KLF4/PODXL signaling pathway assumes an irreplaceable role in tumorigenesis, invasion and metastasis of human GC and PODXL serves as an independent prognostic indicator for GC patients.

**Electronic supplementary material:**

The online version of this article (10.1007/s10120-018-0833-y) contains supplementary material, which is available to authorized users.

## Introduction

Gastric cancer (GC), a leading cause of cancer-related death, is the fifth most common type of cancer worldwide [1]. In China, owing to the ageing and sociodemographic changes and substantial population, the morbidity of GC ranks the second [2], with massive advanced GC. Despite of the use of chemotherapy and surgical resection, the long-term survival remains unsatisfactory on account of recurrence and metastasis. Currently, effective comprehensive treatment, which is able to heal the advanced GC, is not obtainable. Thus, it is of paramount importance to figure out the molecular mechanisms leading to invasion and metastasis of GC.

PODXL, an anti-adhesive transmembrane glycoprotein, is a member of CD34 family, which is initially revealed in podocytes of renal glomeruli. Subsequent study showed that PODXL can also be identified in vascular endothelia [3], hematopoietic progenitors [4] and neurons [5]. Previous study showed that PODXL was involved in cell adhesion of kidney cells via affecting the transepithelial resistance and modifying the distribution of junctional proteins [6]. Recent studies have suggested that the role of PODXL may be of pivotal importance in the development and progression of various cancers [7–9]. It was first revealed that PODXL can be detected as a stem cell marker in malignant cells of testicular cancer [7]. Thereafter, PODXL expression was reported to be associated with tumor aggression and adverse prognosis in several cancers, such as, urothelial bladder cancer [8], colorectal cancer [10], pancreatic cancer [11], hepatocellular carcimoma [9] and GC [12].Emerging evidence revealed that the altered expression of PODXL was implicated with migration, epithelial-mesenchymal transition (EMT), invasion and metastasis of cancers [11, 13, 14]. Lin et al. [13] demonstrated that the level of PODXL was closely correlated with motility and invasiveness of breast cancer cells via activating the Rac1/Cdc42/cortactin signaling. Enforced expression of PODXL increased the formation and activation of invadopodia, resulting in the tumor invasion and metastasis. Additionally, it was found that the altered expression of PODXL lead to the molecular changes (e.g., vimentin, E-cadherin, collagen I) associated with TGF-β induced EMT of human lung adenocarcinoma cells [14], demonstrating the significant role of PODXL in the EMT and metastasis of cancers. All of above clinical and experimental evidences are in favor of the oncogenic role of PODXL in the development of human cancers. However, little is known about the precise functions and underlying mechanisms of PODXL in GC EMT, invasion and metastasis. Moreover, the molecular mechanisms regulating PODXL remain unclear.

Krüppel-like factor 4 (KLF4), a conserved zinc finger transcription factor of Krüppel-like factor family, is a key regulator in maintaining the integrity of progression of cell cycle [15]. Mounting evidences from both experimental and clinical data have suggested that KLF4 functioned as a tumor suppressor in cancers and played a critical role in tumor differentiation, EMT, invasion and metastasis [16, 17]. Interestingly, the villin-positive gastric progenitor cells of transgenic mice with KLF4 inactivation contributed to the transformation of gastric mucosa and then lead to the tumorigenesis in the antrum in mice, which further verified the prominent role of KLF4 in the development of GC [18]. Additionally, recent study indicated that miR-103 can enhance the proliferation and metastasis of GC by repressing KLF4 expression [19]. While, the underlying mechanism that how KLF4 regulates the EMT and metastasis of GC is still unknown.

In our current study, we determined to explore the role of PODXL in the EMT and metastasis of GC and demonstrate the regulatory function of KLF4 in PODXL expression and function. Our study concerning KLF4/PODXL signaling pathway might be conducive to better understand the process of EMT, invasion and metastasis of GC, and provide an independent prognostic indicator and therapeutic target for GC.

## Methods

### Patients and specimens

32 paired fresh GC and surrounding normal tissues were collected after radical gastrectomy in the Shanghai General Hospital and were stored at − 80 °C refrigerator for RNA extraction. 57 paired GC and surrounding normal tissues were obtained from patients diagnosed with GC at General Surgery Department of Shanghai General Hospital from 2013 to 2014. All specimens, preparing to construct tissue microarray (TMA), were paraffin-embedded, then validated by hematoxylin and eosin (H&E) staining and finally examined by two independent pathologists. The final TMA covered 54 GC tissues and 57 adjacent normal tissues. All patients enrolled had yet to receive radiotherapy and chemotherapy before surgery. Clinicopathological characteristics, diagnosed and confirmed by two independent pathologists, were presented in Table 1. All written informed consents were obtained before enrolling in the study. And the study was ratified by the Ethical Committee for Clinical Research of Shanghai General Hospital.

**Table 1:** Correlation between PODXL expression and clinicopathological parameters in GC (*n* = 54)

Parameters	Category	No.	PODXL expression		*p*
			Negative	Strong	
Age					0.821339
	< 65	26	11	15	
	≥ 65	28	11	17	
Gender					0.753143
	Male	38	16	22	
	Female	16	6	10	
T stage^a^					0.025968*
	T2	5	4	1	
	T3	21	11	10	
	T4	28	7	21	
*N* stage					0.020323*
	*N*0	11	7	4	
	*N*1	12	8	4	
	*N*2	13	3	10	
	*N*3	18	4	14	
UICC stage^b^					0.015122*
	I&II	17	11	6	
	III&IV	37	11	26	
Nerve invasion					0.117946
	Yes	29	9	20	
	No	25	13	12	
Vessel invasion					0.510348
	Yes	29	13	16	
	No	25	9	16	
Differentiation					0.000867***
	Well	3	3	0	
	Moderate	19	12	7	
	poor	32	7	25	
Tumor size	≤ 3	23	8	15	0.442764
	> 3	31	14	17	

*p < 0.05

***p < 0.001

^a,b^No T1 stage or M1 patients was appeared in GC tissues microarray

### Cell lines

The human gastric cancer cells, including AGS, SGC7901, MGC803, BGC823, MKN45, MKN28, HGC27, were obtained from the Type Culture Collection of the Chinese Academy of Science (Shanghai, China). All cells were cultured in 1640 medium mingled with 10% fetal bovine serum (Gibco, Carlsbad, CA) and 1% penicillin–streptomycin. All the cells were maintained at 37 °C filled with 5% CO_2_.

### The Oncomine and the Kaplan–Meier Plotter

The mRNA expression of PODXL in GC tissues and normal mucosae was acquired from Oncomine (http://www.oncomine.org). The results of prognostic analysis of PODXL and KLF4 were obtained from Kaplan–Meier Plotter (http://www.kmplot.com).

### RNA extraction and quantitative real-time PCR(qRT-PCR)

Based on the protocol previously described [20], the total RNA of GC cell lines, tissues and adjacent normal tissues, was extracted and then transcribed into complementary DNA, respectively. The ΔΔCt method was performed to evaluate relative targeted genes levels. The specific primers used in study were the same as that depicted previously [20]. Each result was carried out in triplicate.

### Transient transfection and lentiviral transduction

The pcDNA3.1-KLF4 plasmid and negative control vector were synthesized from Obio Technology Co.,Ltd (Shanghai, China). The oligonucleotides were performed employing Lipofectamine™ 2000 (Invitrogen, USA) as described previously [21]. The short hairpin RNA (shRNA) targeting PODXL was bought form Genechem Co., Ltd. (Shanghai, China). Sense: TATCAGTGAGATCAATTTC, Antisense: GAAATTGATCTCACTGATA. For lentivirus transfections, GC cells in logarithmic growth phase, cultured in 6-well plates, were transfected with lenti-shPODXL-virus or lenti-control virus. Thereafter, the GC cells with lentiviral transduction were purified with puromycin (Invitrogen, USA).

### Nude mice xenograft models

To establish subcutaneous tumor, about 7 × 10^6^ cells were subcutaneously injected into a nude mouse. 28 days after injection, all mice were sacrificed, and then the xenograft tissues were excised. Then the tumor weight and volume were measured. The following formula was used to calculated tumor volume: volume = 0.5 × width^2^ × length. For liver metastatic assay, 5 × 10^6^ cells were injected via ileocolic vein as we did previously [21]. The mice were killed and the livers were then removed after 21 days’ inoculation or when they were moribund. The tissues were then paraffin-embedded and the slides finally validated by hematoxylin and eosin (H&E) staining. All mice were used in line with the guidelines of Institutional Animal Care of Shanghai General Hospital, and all efforts were employed to weaken animal suffering.

### Immunohistochemistry (IHC)

After deparaffinization in xylene, the microarray of GC were rehydrated in the gradient ethanol. Then the microarray, prepared for antigen retrieval, was boiled in 10 mM sodium citrate buffer (PH 6.0) for 4 min. After endogenous peroxidase activity of the slide was blockaded by hydrogen peroxide for 10 min, the slides were covered with primary antibody: PODXL (1:200, Santa cruz, CA, USA), KLF4 (1:200, Santa cruz, CA, USA) at 4 °C overnight. Next, incubated with secondary antibody for half an hour at room temperature, the microarrays were then soaked with diaminobenzidine and Mayer’s hematoxylin. Finally, the microarray was dehydrated in the gradient ethanol and covered with cover slips. The scores of GC specimens were undertaken by two independent pathologist as reported previously [20].

### Chromatin immunoprecipitation (ChIP)

SimpleChIP enzymatic Chromatin IP kit(magnetic beads) (Cell Signaling Technology, USA) was used for ChIP assay. 293T cells (4*10^6^), transfected with vector or KLF4, were cross-linked, quenched with glycine and then lysed by sonication. Next, immunoprecipitation was performed with the antibody against KLF4 (R&D, #AF3640), and IgG was applied as a normal control. Finally, precipitated DNA was amplified by PCR and examined for the presence of PODXL promoter sequence. And the primers were used as follows: forward: 5′-TCTTGCCACCAGGACACCTA-3′, reverse: 5′-TACCTCTTCCCAGACCCAAT-3′.

### Statistical analysis

SPSS 21 statistical software (SPSS Inc., USA) was carried out to analyse all data. The significance of paired and unpaired continuous variables was calculated by Student’s *t* test and Mann–Whitney *U* test. The significances between targeted genes expression and clinicopathological features were determined appropriately by Fisher’s exact test and the *χ*^2^ test. Kaplan–Meier method and log-rank test were performed for survival curves and differences, respectively. The HR with 95% confidence interval and significance between individual factors and overall survival (OS), disease-free survival (DFS) were performed via Cox proportional hazards regression. *P* value < 0.05 was considered to be significant.

## Results

### PODXL was upregulated significantly in human GC

**Fig. 1:**
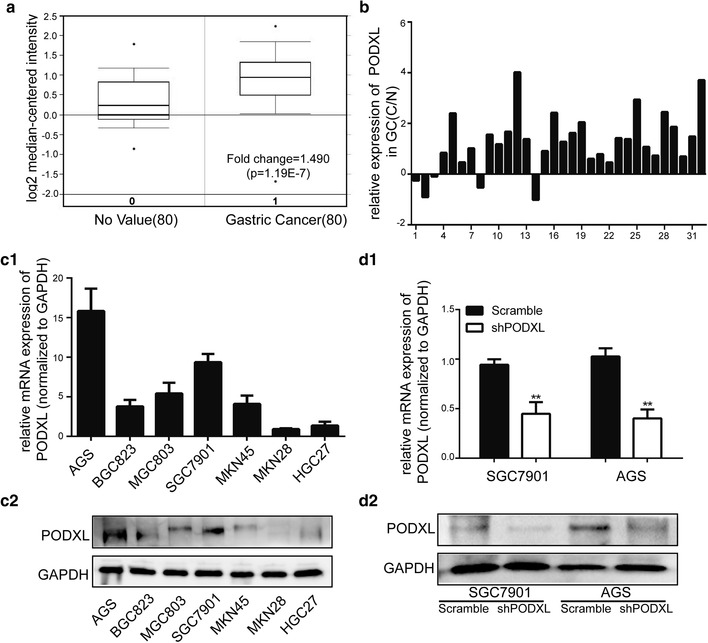
PODXL expression in GC tissues and GC cell lines. **a** Oncomine data showed that PODXL expression was elevated in GC tissues as compared with gastric normal tissues. **b** The expression of PODXL in 32 tissues and surrounding normal tissues was detected by qRT-PCR. PODXL level was higher in 27 (84.38%) GC tissues. **c1, c2** The level of PODXL in GC cell lines was determined by qRT-PCR and western blot. The expression of PODXL in SGC7901 and AGS was relatively high. **d1, d2** The expression of PODXL in SGC7901 and AGS transfected with negative control or shPODXL was measured by qRT-PCR and western blot. Mean ± SEM was applied for analysis

Public data (Oncomine) were utilized to detect the level of PODXL in GC and adjacent normal tissues. And it was found that PODXL was significantly overexpressed in GC tissues (Fig. 1a). Thereafter, we sought to verify the expression of PODXL in 32 paired GC and surrounding normal tissues. The result of qRT-PCR revealed PODXL was significantly high in GC tissues (Fig. 1b). To examine PODXL expression in GC cell lines, qRT-PCR and western blot were performed, in which outcomes demonstrated that PODXL level was relatively elevated in SGC7901 and AGS cell lines as compared with others (Fig. 1c1, c2). Subsequently, the levels of PODXL in SGC7901 and AGS were selected for knockdown to further investigate the biological processes. The qRT-PCR and western blot outcomes revealed that PODXL expression was significantly blockaded in PODXL knockdown group (Fig. 1d1, d2).

### Association between PODXL and its pathological features in GC

**Fig. 2:**
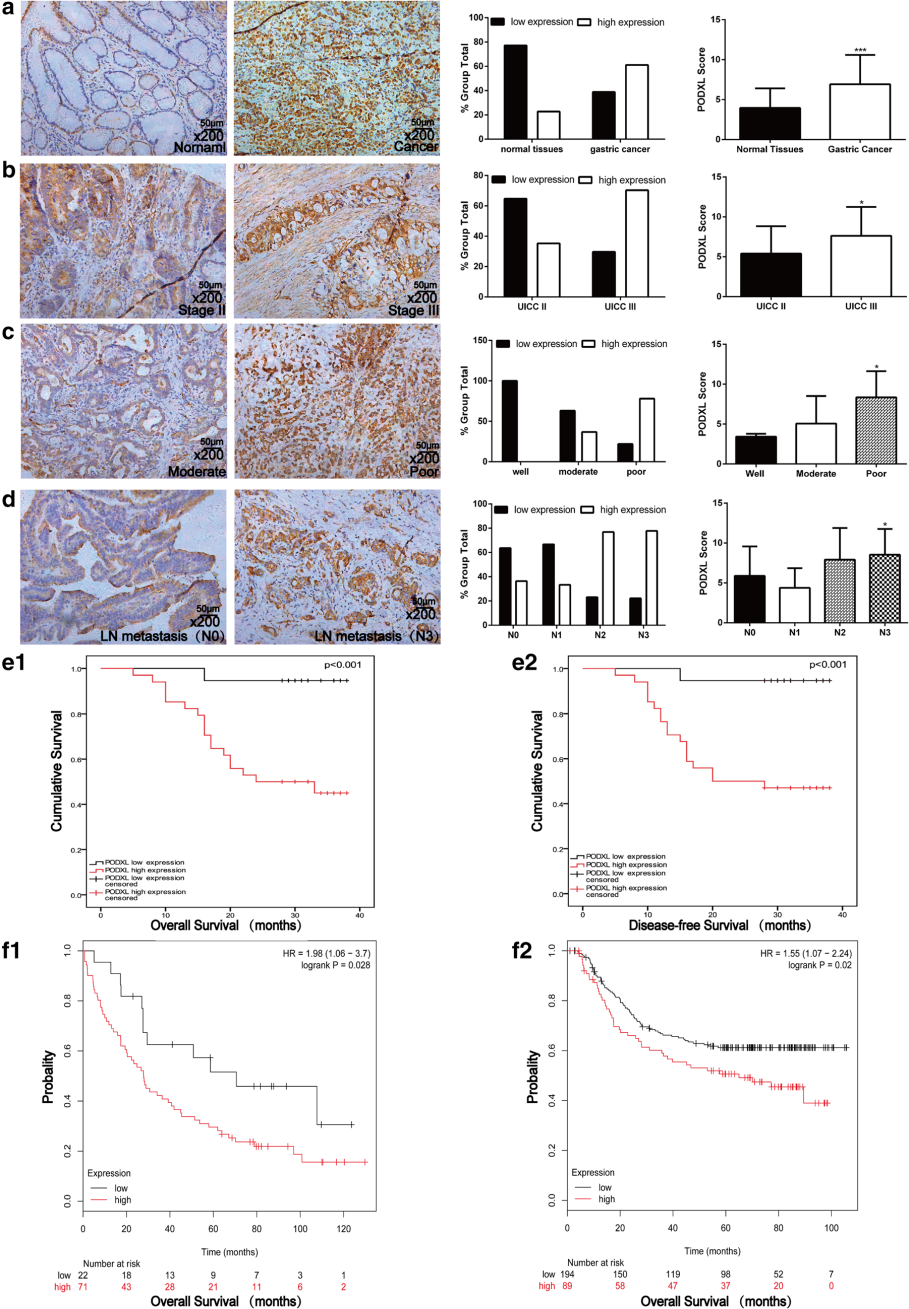
The level of PODXL in GC tissues microarray was determined by immunohistochemistry. **a** Representative images of PODXL level in normal gastric mucosa and GC specimens. The expression of PODXL was negative in normal gastric mucosa and positive in gastric cancer specimens. **b** The expression of PODXL was positively associated with tumor stage, and the representative images of stage II and III were presented. **c** The expression of PODXL was positively associated with tumor differentiation, and the representative images of grade II and III were presented. **d** The expression of PODXL was positively associated with tumor lymph node metastasis, and the representative images of tumor with or without lymph node metastasis were presented. **e1, e2** Patients with lower level of PODXL had a better OS (*p* < 0.001) and DFS (*p* < 0.001) in GC tissues. **f1, f2** The data (GSE51105 for F1, GSE62254 for F2) from Kaplan–Meier Plotter revealed that patients with decreased PODXL showed a longer OS (*p* < 0.05)

To further determine PODXL expression and investigate the association between PODXL and its clinical features in human GC specimens, GC microarray containing 54 cases of primary GC and 57 adjacent normal tissues was used for immunohistochemistry. We observed that the cytoplasm of tumor cells was evenly PODXL-positive staining and the tumor stroma was PODXL-negative. Among the 54 GC specimens, 32(59.3%) cases showed positive staining (Fig. 2a). Further analysis demonstrated that PODXL expression was significantly correlated with tumor stage (Table 1), indicating that PODXL was elevated in advanced tumor stage (Fig. 2b). Moreover, PODXL was overexpressed in poor differentiation (Fig. 2c) and increasing PODXL expression was significantly associated with worse tumor differentiation (Table 1). Additionally, the level of PODXL in GC tissues with lymph node metastasis was obviously higher in comparison with one without lymph node metastasis(Fig. 2d) and the statistical difference was significant (Table 1). In view of other clinical parameters, our study demonstrated that upregulated PODXL expression was significantly associated with T stage, while no correlation was observed between PODXL level and age, gender, nerve invasion, vessel invasion (Table 1). Our findings strongly illustrate that PODXL level plays an irreplaceable role in GC development and progression.

**Fig. 3:**
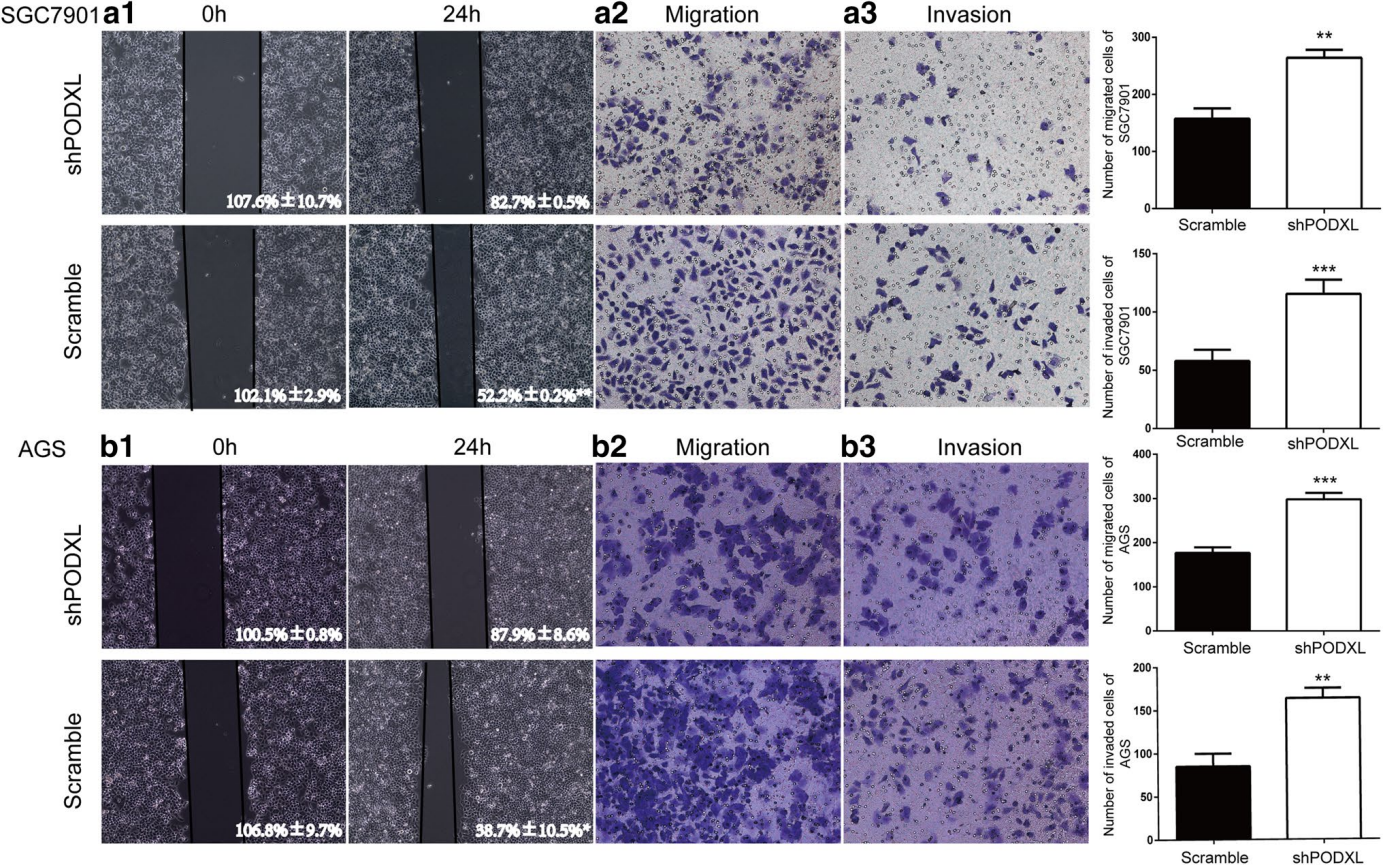
The influence of PODXL level on GC cell lines migration and invasion. **a1, b1** Wound healing assay was used to detect the change of migration of cells. The migrated ability of SGC7901 and AGS was reduced in PODXL knockdown group. Transwell assays were used to detect the change of migration and invasion of cells. **a2, b2** The migrated ability of SGC7901 and AGS was reduced in PODXL blockage group. **a3, b3** The invaded ability of SGC7901 and AGS was reduced in shPODXL group (*p < 0.05, **p < 0.01, ***p < 0.001)

### PODXL overexpression predicted poorer clinical results in GC

To better analyze that whether PODXL expression can be regarded as a valuable biomarker for the prognosis of GC, Cox proportional hazard model was performed (supplement table 1). Univariate analysis revealed that pN stage, UICC stage and PODXL level were significantly correlated with OS and DFS of GC. Further multivariate analysis was carried out using the above factors, and the outcome suggested that the correlation between high level of PODXL and OS (HR = 8.717, 95% CI:1.110–68.437, *p* = 0.039), DFS (HR = 8.923, 95%CI:1.138–69.976, *p* = 0.037) was significant (supplement table 1).

To figure out the relationship between PODXL expression and prognosis of GC, Kaplan–Meier method with log-rank test and bioinformation from Kaplan–Meier Plotter were undertaken. The final results of microarray showed that patients with lower level of PODXL had a better OS and DFS as compared with these with higher level of PODXL (Fig. 2e1, e2). Additionally, the data of Kaplan–Meier Plotter further verified that PODXL level was significantly correlated with OS in patients of GC (Fig. 2f1, f2). All these findings further suggest that PODXL is of vital significance in prognosis of patients of GC and high level of PODXL may serve as an independent biomarker for poor prognosis of GC patients.

### Altered PODXL expression affected migration and invasion in vitro

**Fig. 4:**
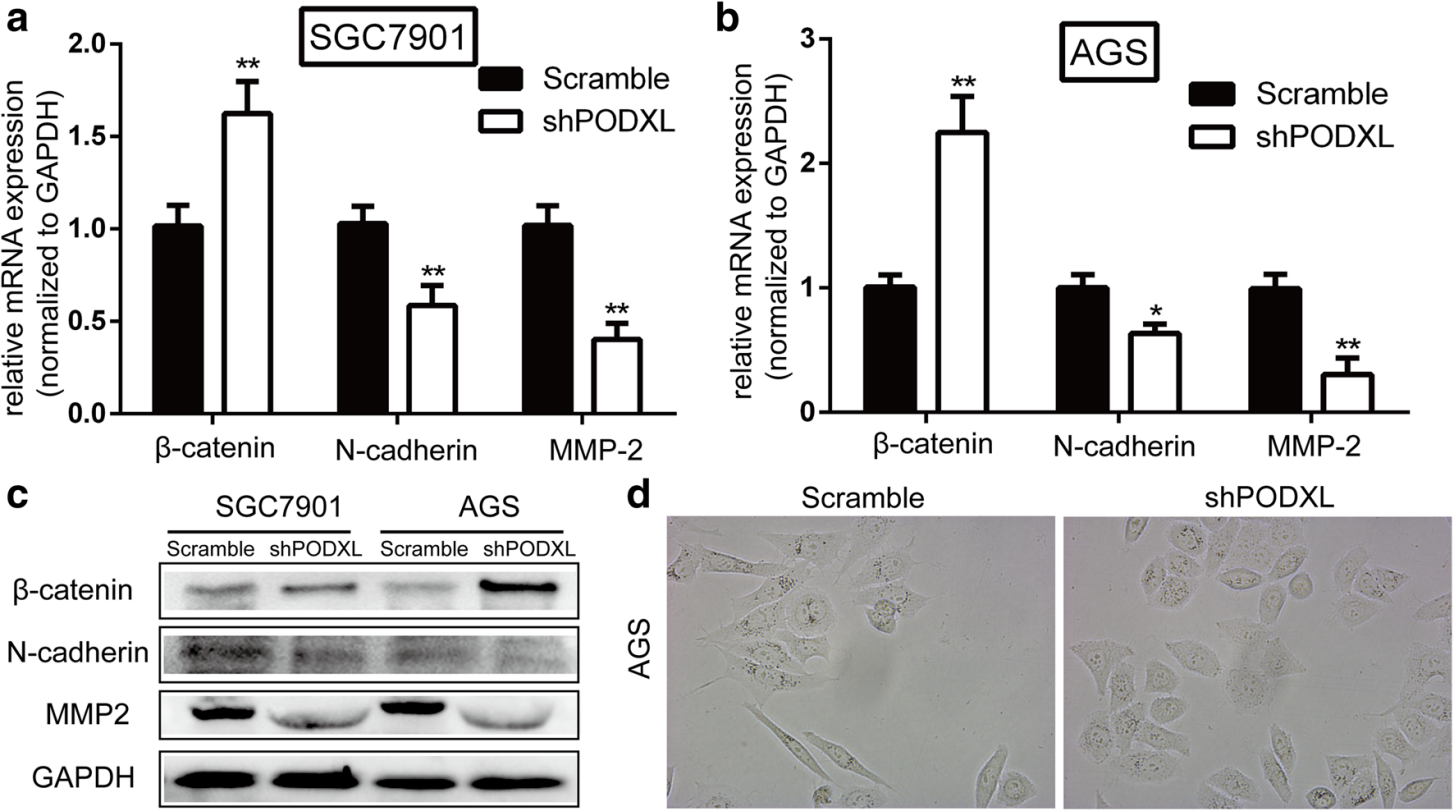
The influence of PODXL level on EMT and metastasis. **a, b** qRT-PCR was applied to detect the change of EMT-associated and metastatic genes. The level of N-cadherin and MMP-2 was reduced and the level of β-catenin was elevated when blockading PODXL expression. c Western blot was applied to detect the change of EMT-associated and metastatic genes. The level of N-cadherin and MMP-2 was reduced and the level of β-catenin was elevated when blockading PODXL expression. **d** Representative images showed that AGS with PODXL knockdown exhibited a typical epithelial phenotype, whereas the scramble group exhibited a typical mesenchymal phenotype

To determine the function of altered PODXL expression on migration of GC cells, SGC7901 and AGS transfected with lentiviral shPODXL and scramble vector were applied. The lentiviral transducted cells were scratched, washed and cultured at 37 °C for additional 24 h. And the wound healing assays indicated that knockdown of PODXL expression delayed spreading of both GC cells (Fig. 3a1, b1). Further migration assay showed that blockage of PODXL expression attenuated the migration ability of SGC7901 and AGS (Fig. 3a2, b2). Similarly, low level of PODXL decreased the invasiveness of SGC7901 and AGS (Fig. 3a3, b3). And these findings were additionally supported by the change of the biomarker associated with invasion and metastasis. Western blot demonstrated that the level of MMP-2 in SGC7901 and AGS with lower PODXL expression was reduced (Fig. 4a–c). Taken together, our data illustrate that downregulation of PODXL could inhibit the migration and invasion of GC cells in vitro.

### Altered PODXL expression affected EMT in vitro and tumorigenesis in vivo

**Fig. 5:**
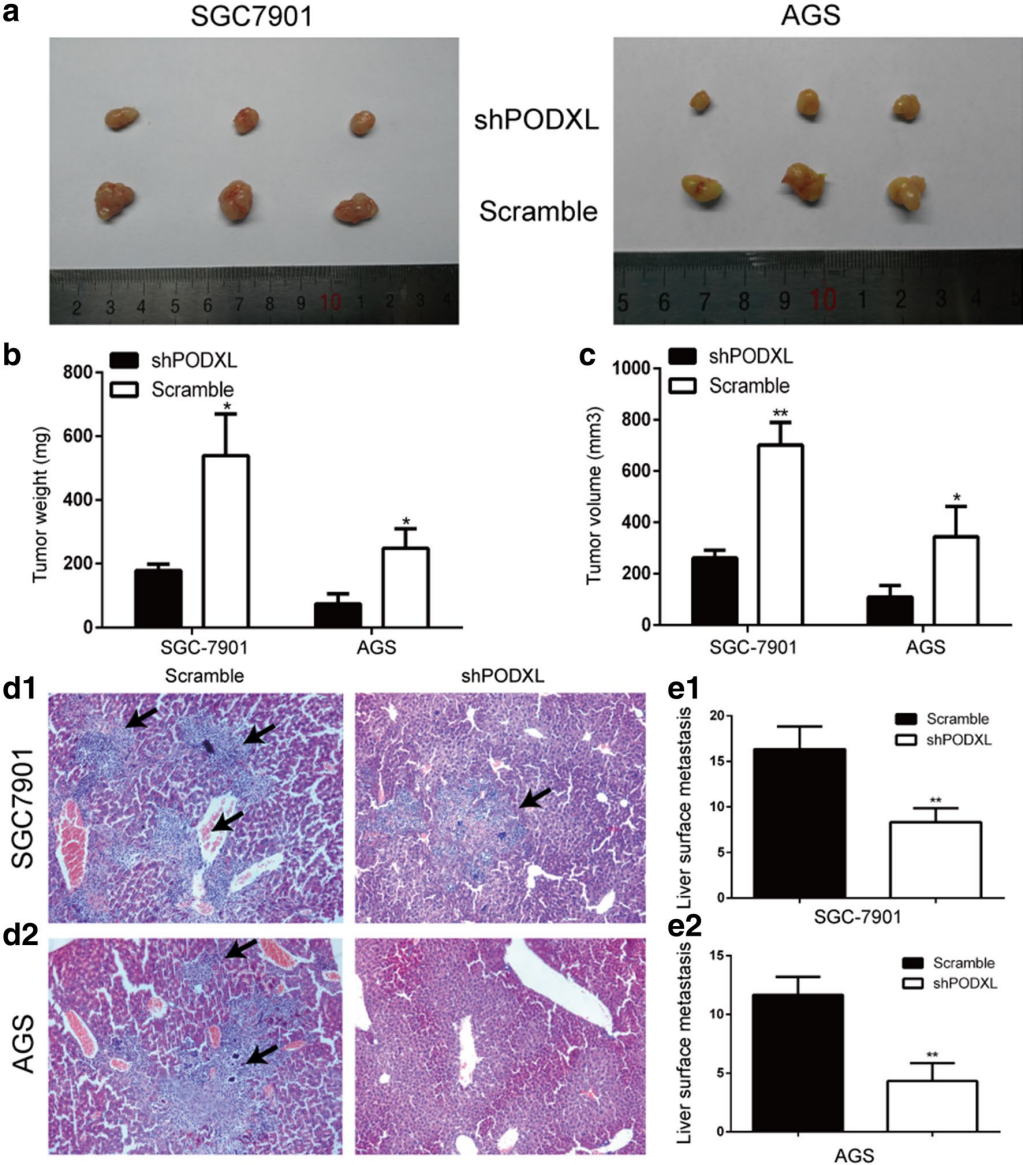
Influence of altered PODXL level on GC tumorigenicity and metastasis in vivo. **a** representative images of shPODXL and scramble group. The tumors of scramble group were larger than PODXL knockdown group as measured by tumor weight (**b**) and volume (**c**). **d1, d2** The hematoxylin and eosin (HE) staining of liver. Blockage of PODXL inhibited the liver metastasis in nude mice as compared with scramble group. **e1, e2** The number of liver surface metastasis of scramble group was higher than that of shPODXL group

To determine the function of altered PODXL expression on EMT of GC cells, SGC7901 and AGS were transfected with lentiviral shPODXL and scramble vector. Our study revealed that decreased mRNA of PODXL in SGC7901 and AGS cells led to the reduction of N-cadherin mRNA but increase of β-catenin mRNA significantly (Fig. 4a, b). Consistent with these, reduction of N-cadherin protein and increase of β-catenin protein were observed when blockading PODXL in SGC7901 and AGS (Fig. 4c). Furthermore, we found that AGS, typically having mesenchymal phenotype, transferred into epithelial phenotype when PODXL level was downregulated (Fig. 4d). The above results clearly demonstrated that the altered PODXL level have an effect on the phenotype of epithelial or mesenchymal of GC cells. Consistent with the functions of altered PODXL level on migration and invasion of GC cells *in vitro*, SGC7901 and AGS transfected with shPODXL significantly inhibited tumor growth in nude mice in comparison with the control group (Fig. 5a–c). Moreover, downregulated expression of PODXL in SGC7901 and AGS can inhibit the liver metastasis in nude mice (Fig. 5d1,d2,e1,e2). To sum up, our outcomes distinctly show that PODXL is an oncogene and blockage of PODXL can prevent the tumor growth and metastasis of GC *in vivo*.

### The correlations and interaction between KLF4 and PODXL in GC

**Fig. 6:**
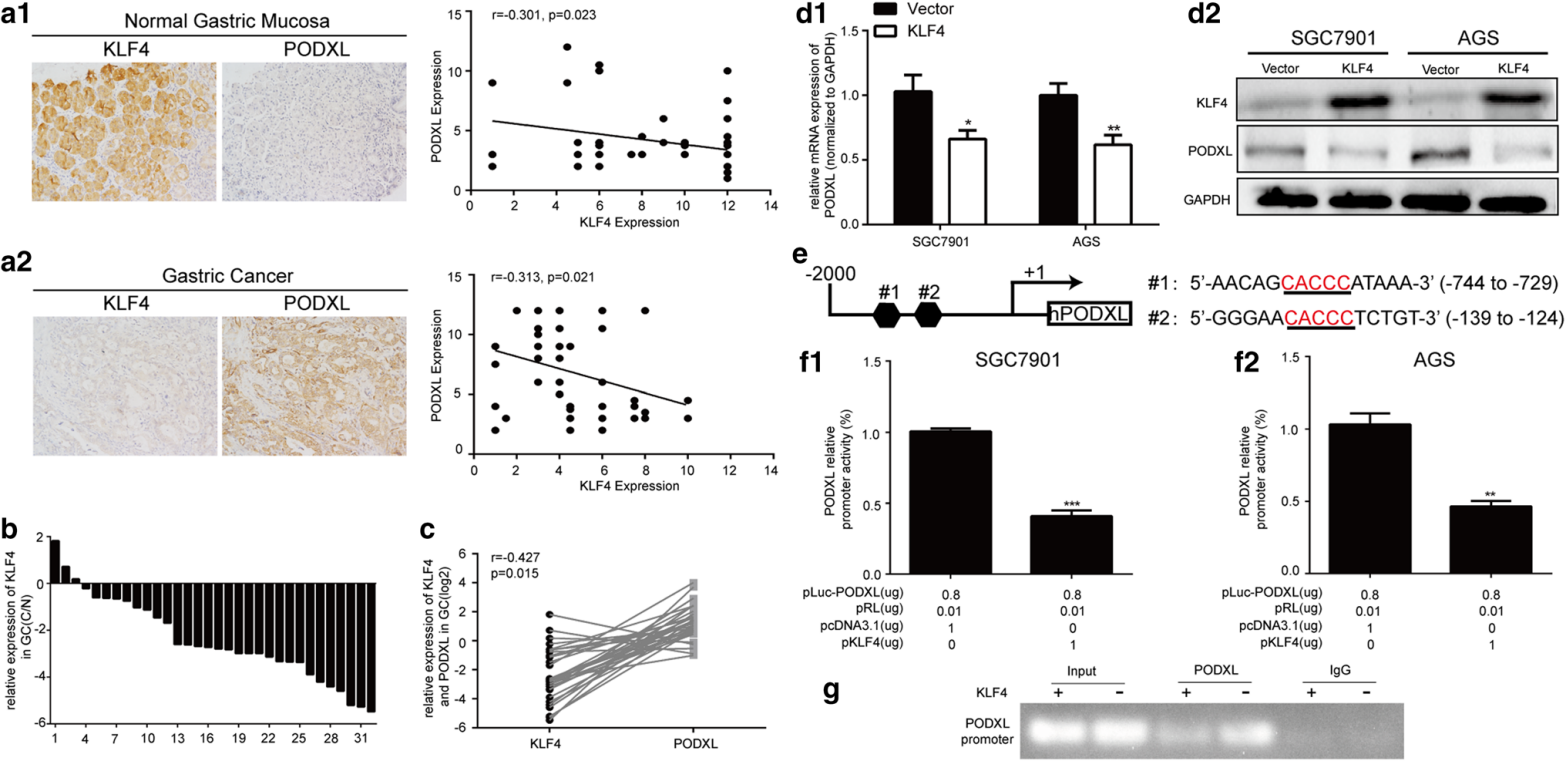
The correlation between KLF4 and PODXL in GC. The relationships between KLF4 and PODXL in normal gastric mucosa (**a1**) (200x) (*r* = − 0.310, p = 0.023) and GC tissues (**a2**) (200x) (*r* = − 0.313, *p* = 0.021), detected by IHC, were negative. **b** The mRNA expression of KLF4 in 32 GC tissues. **c**The correlation between KLF4 and PODXL mRNA in 32 GC tissues was also negative (*r* = − 0.427, *p* = 0.015). The mRNA (**d1**) and protein (**d2**) level of PODXL were attenuated when KLF4 level was enforced in SGC7901 and AGS. **e** The potential binding sites for KLF4 at the 5′UTR of PODXL. **f1, f2** The effect of KLF4 level on PODXL promoter activity. Luciferase assay revealed that KLF4 could suppress the promoter activities of PODXL in SGC7901 and AGS. **g** Chromatin immunoprecipitation (ChIP) assay followed by PCR showed that KLF4 directly binds PODXL promoter

To figure out the underlying molecular mechanisms of PODXL overexpression, immunohistochemistry was first performed to detect the expression of KLF4 and PODXL in GC microarray. Interestingly, we found that the level of KLF4 was higher in gastric mucosa opposite to the lower expression of PODXL in normal gastric mucosa. Consistently, the staining of KLF4 was weaker in the most GC tissues, while PODXL staining was strongly positive. Further analysis revealed that the correlations between KLF4 and PODXL in gastric mucosa and GC tissues were negative with statistical significance (Fig. 6a1, a2). Additionally, qRT-PCR demonstrated that KLF4 level was lower in GC tissues (Fig. 6b), and the correlation between KLF4 and PODXL mRNA in GC tissues was also negative and statistically significant (Fig. 6c). To provide detailed evidence for the direct correlation between KLF4 and PODXL, we determined the impacts of KLF4 on PODXL expression in human GC cell lines. We discovered that enforced expression of KLF4 in SGC7901 and AGS led to significantly lower PODXL mRNA and protein (Fig. 6d1, d2). Finally, bioinformatics demonstrated that there may be two binding sites of KLF4 in the 5′UTR of PODXL (Fig. 6e). Thus, luciferase assay was performed, and it was found that the relative luciferase activity was decreased in SGC7901 and AGS when transfected with KLF4 and plasmid of 5′UTR of PODXL (Fig. 6f1, f2). Collectively, our findings strongly uncover that KLF4 can negatively regulate PODXL expression in GC cell lines by directly binding to the 5′UTR of PODXL.

## Discussion

Owing to the high economic burden and low public awareness, the morbidity of advanced GC in China is extremely high, which leads to the great mass of cancer-related deaths [1]. Despite of improvement of surgery and chemotherapy for GC, the recurrence and metastasis of advanced GC is still at its peak [22]. Thus, there is an urgent need to figure out the molecular mechanisms of invasion, metastasis and EMT of GC. In our current study, we explored the pivotal roles of KLF4 and PODXL in development and progression of GC and their underlying mechanisms. We found that KLF4 and PODXL constitute a novel signaling pathway, which is irreplaceable in invasion, metastasis and EMT of GC cells. Our findings may have presented an unique perspective on the pathogenesis and aggressive biology of GC.

PODXL, located on chromosome 7q32-q33, is a transmembrane sialomucins of CD34 family [23]. Initially, PODXL was identified in podocytes, vascular endothelia and neurons and PODXL was essential for development of cells [3, 5, 24]. Subsequently, many lines of evidence supported that PODXL occupied a significant role in the development and progression of cancers via acting as an oncogene [7, 25]. First, PODXL was reported to be overexpressed in various cancers [8, 10, 26, 27]. It was demonstrated by Kaprio et al. that PODXL was evaluated to be overexpressed in the vast majority of colorectal cancers [10]. Based on the recent study, the level of PODXL in GC tissues was significantly higher in comparison with normal ones [27]. Furthermore, available articles manifested that PODXL may be an independent prognostic marker in cancers [8, 12, 28]. Boman et al. found that PODXL overexpression was significantly correlated with a low 5-year OS in urothelial bladder cancer and PODXL was an independent prognostic indicator for patients with urothelial bladder cancer [8]. Additionally, mounting evidence illustrated that PODXL level was significantly correlated with clinical features, such as advanced tumor stage, high-grade stage and so forth [10, 11, 29]. Forse et al. revealed that elevated PODXL expression was correlated with high histological grade, large tumor size and so on [29]. In our present study, we demonstrated that PODXL was an oncogene from different angles. In addition, elevated PODXL expression was positively correlated with advanced tumor stage, high-grade stage and lymph node metastasis, which was consistent with the roles of PODXL in other cancers. Taken together, we manifested from molecular and clinical evidence that PODXL could promote GC invasion and metastasis.

The process of invasion and metastasis is complicated and the detailed molecular basis remains to be explored [30]. Emerging evidence suggested that PODXL was involved in the process of invasion, metastasis and EMT in various cancers [13, 14, 23]. Enforced PODXL expression in breast and prostate cancer cell lines promoted the migrated and invasive abilities by increasing expression of MMP-1 and MMP-9, and activating MAPK and PI3K signaling [31]. Moreover, silencing of PODXL level in human lung adenocarcinoma cells decreased the spreading and migration abilities and resulted in the phenotype changes of cells by affecting EMT-associated protein, such as vimentin and E-cadherin. This findings manifested that PODXL was implicated in cell migration and invasion via EMT process [14]. Additionally, recent study also revealed that the deletion of PODXL in breast cancer cells led to inhibition of primary tumor growth and metastasis in vivo [23]. However, little was known concerning the roles of PODXL in the invasion and metastasis of GC cells. In the present study, we found that the migration and invasion ability was attenuated when silencing PODXL expression in GC cell lines. Further assays revealed that silencing PODXL level in GC cell lines led to reduction of MMP-2 expression, which could give rise to the decrease of migration and invasion of GC cells. Additionally, we observed that knockdown of PODXL in GC cell lines contributed to the acquisition of epithelial phenotype by upregulation of epithelial marker (β-catenin) and downregulation of mesenchymal marker (N-cadherin). Furthermore, we proved that silencing PODXL in GC cell lines led to the inhibition of tumor growth and metastasis in nude mice, consistent with above study. Thus, we demonstrated first that PODXL can play a critical role in migration, invasion and EMT of GC.

In view of the significant role of PODXL in GC migration, invasion and EMT, we further explored the underlying molecular mechanisms regulating PODXL overexpression in GC. Our current study verified that KLF4, a critical transcriptional factor that had been reported to be involved in the development of different cancers, was the upstream target of PODXL. Supporting evidences were provided from diverse angles: First, the correlation between KLF4 and PODXL expression in GC microarray was negative. Second, the mRNA level of KLF4 was negatively associated with that of PODXL in GC tissues. Additionally, elevated expression of KLF4 directly contributed to decrease of PODXL. Moreover, further bioinformatics and luciferase reporter assay revealed that KLF4 could directly bind to the 5′UTR of PODXL and then negatively regulate the level of PODXL. Therefore, we concluded that there was a novel KLF4/PODXL signaling pathway contributing to GC invasion and metastasis from clinical and mechanistic evidence. Interestingly, our previous and other studies illustrated that PODXL can also be regulated by a great many of miRNAs [20, 32, 33]. And it was necessary to figure out possibility of the cross-talks between miRNAs and KLF4 in regulating PODXL expression, contributing to aggression of GC.

To sum up, our study explored the vital role of PODXL in EMT, progression and metastasis of GC and the molecular mechanisms between KLF4 and PODXL. Collectively, our current study not only manifested that PODXL may serve as an independent prognostic indicator for GC, but also revealed a novel KLF4/PODXL signaling pathway in GC, which may be a promising molecular target for the treatment of GC.

## Electronic supplementary material

Below is the link to the electronic supplementary material.

Supplementary material 1 (DOC 10678 KB)
