# Supplementary material for: PODXL, negatively regulated by KLF4, promotes the EMT and metastasis and serves as a novel prognostic indicator of gastric cancer
Source: Gastric Cancer. 2018 May 10;22(1):48–59. doi: 10.1007/s10120-018-0833-y (PMC6314994; doi:10.1007/s10120-018-0833-y)
Supplement: Supplementary file 1 — Supplementary material 1 (DOC 10678 KB) [file 10120_2018_833_MOESM1_ESM.doc]

**Supplement Table.1 univariate and multivariate analysis for GC overall survival (OS) and disease-free survival (DFS)**

| Parameters | No. | Overall survival | | | | Disease-free survival | | | |
| --- | --- | --- | --- | --- | --- | --- | --- | --- | --- |
|  |  | unvariate analysis | | Multivariate analysis | | unvariate analysis | | Multivariate analysis | |
|  |  | HR(95%CI) | P | HR(95%CI) | p | HR(95%CI) | P | HR(95%CI) | p |
| Age |  |  | 0.732 |  |  |  | 0.826 |  |  |
| ＜65 | 26 | 1.171  (0.475-2.886) |  |  |  | 1.106  (0.449-2.723) |  |  |  |
| ≥65 | 28 |  |  |  |  |  |  |  |  |
| Gender |  |  | 0.695 |  |  | 0.819  (0.295-2.275) | 0.702 |  |  |
| Male | 38 | 0.815  (0.293-2.265) |  |  |  |  |  |  |  |
| Female | 16 |  |  |  |  |  |  |  |  |
| T stage |  |  | 0.107 |  |  |  | 0.101 |  |  |
| T2 | 5 | 1.924  (0.868-4.264) |  |  |  | 1.957  (0.878-4.365) |  |  |  |
| T3 | 21 |  |  |  |  |  |  |  |  |
| T4 | 28 |  |  |  |  |  |  |  |  |
| N stage |  |  | 0.005 |  | 0.301 |  | 0.005 |  | 0.296 |
| N0 | 11 | 2.056  (1.240-3.409) |  | 1.493  (0.699-3.191) |  | 2.052  (1.242-3.390) |  | 1.494  (0.703-3.175) |  |
| N1 | 12 |  |  |  |  |  |  |  |  |
| N2 | 13 |  |  |  |  |  |  |  |  |
| N3 | 18 |  |  |  |  |  |  |  |  |
| UICC stage |  |  | 0.034 |  | 0.803 |  | 0.035 |  | 0.811 |
|  |  | 4.882  (1.123-21.217) |  | 1.312 |  | 4.830  (1.113-20.948) |  | 1.297  (0.153-10.990) |  |
| II  III | 17  37 |  |  | (0.156-11.039) |  |  |  |  |  |
| Nerve invasion |  |  | 0.165 |  |  |  | 0.164 |  |  |
| Yes  No | 29  25 | 0.503 |  |  |  | 0.503 |  |  |  |
|  |  | (0.191-1.325) |  |  |  | (0.191-1.324) |  |  |  |
| Vessel invasion |  |  | 0.776 |  |  |  | 0.770 |  |  |
| Yes | 29 | 0.876  (0.352-2.179） |  |  |  | 0.873  (0.351-2.170) |  |  |  |
| No | 25 |  |  |  |  |  |  |  |  |
| Differentiation |  |  | 0.162 |  |  |  | 0.154 |  |  |
| Well | 3 | 1.922  (0.769-4.804） |  |  |  | 1.951  (0.778-4.891) |  |  |  |
| Moderate  poor | 19  32 |  |  |  |  |  |  |  |  |
| Tumor size |  |  | 0.699 |  |  |  | 0.758 |  |  |
| ≤3 | 23 | 0.837  (0.340-2.061) |  |  |  | 0.868  (0.353-2.136) |  |  |  |
| ＞3 | 31 |  |  |  |  |  |  |  |  |
| PODXL |  |  | 0.012 |  | 0.039 |  | 0.012 |  | 0.037 |
| Low | 20 | 13.189  (1.758-98.948) |  | 8.717  (1.110-68.437） |  | 13.404  (1.786-100.580) |  | 8.923  (1.138-69.976) |  |
| high | 34 |  |  |  |  |  |  |  |  |

HR：hazard ratio; CI: confidence interval

*p<0.05 indicate that the 95% CI of HR was not included


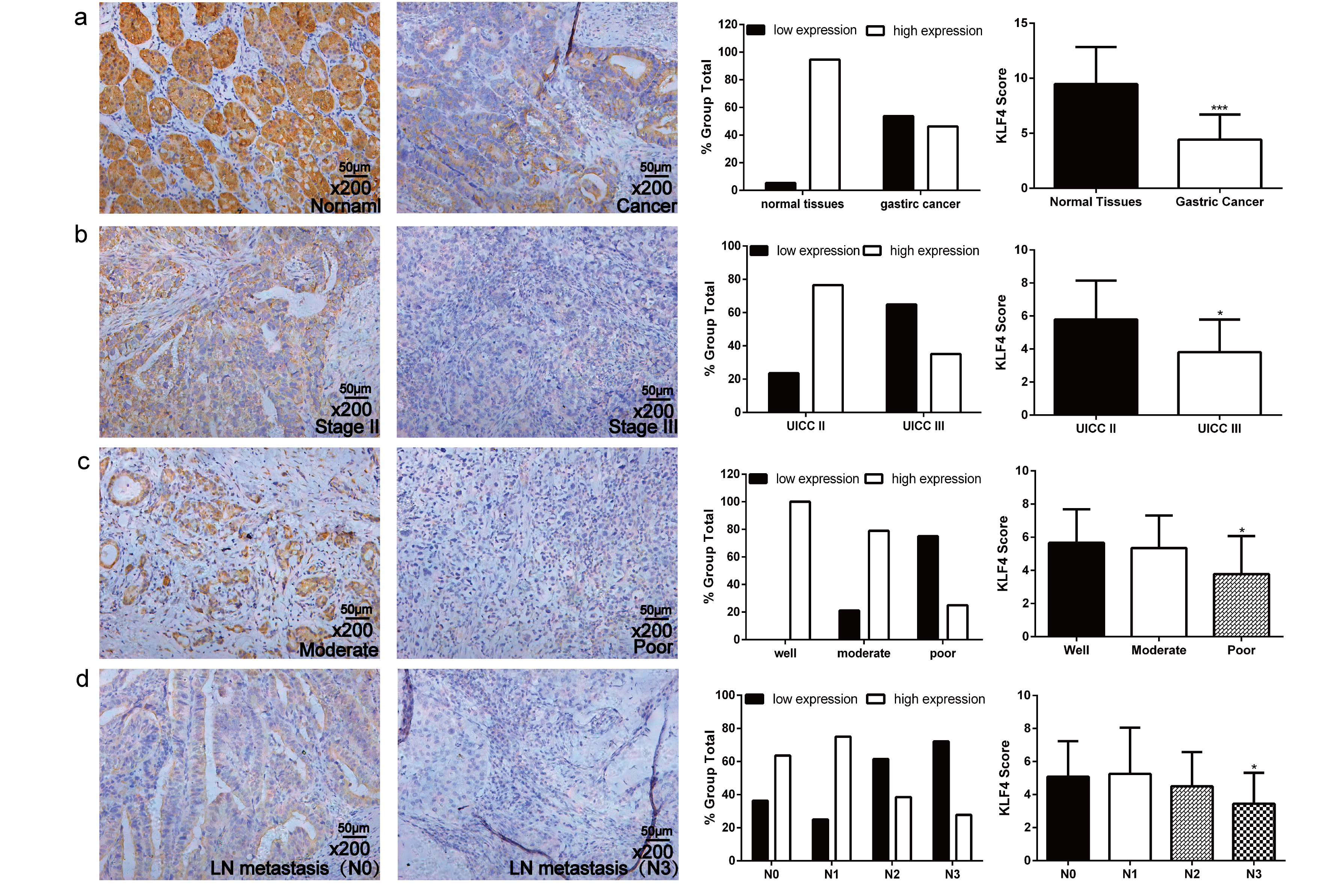


**S1. The level of KLF4 in GC tissues microarray was determined by immunohistochemistry.** (**a**) Representative images of KLF4 level in normal gastric mucosa and GC specimens. The expression of KLF4 was positive in normal gastric mucosa and negative in gastric cancer specimens. (b) The expression of KLF4 was negatively associated with tumor stage, and the representative images of stage II and III were presented. (**c**) The expression of KLF4 was negatively associated with tumor differentiation, and the representative images of grade II and III were presented. (**d**) The expression of KLF4 was negatively associated with tumor lymph node metastasis, and the representative images of tumor with or without lymph node metastasis were presented.


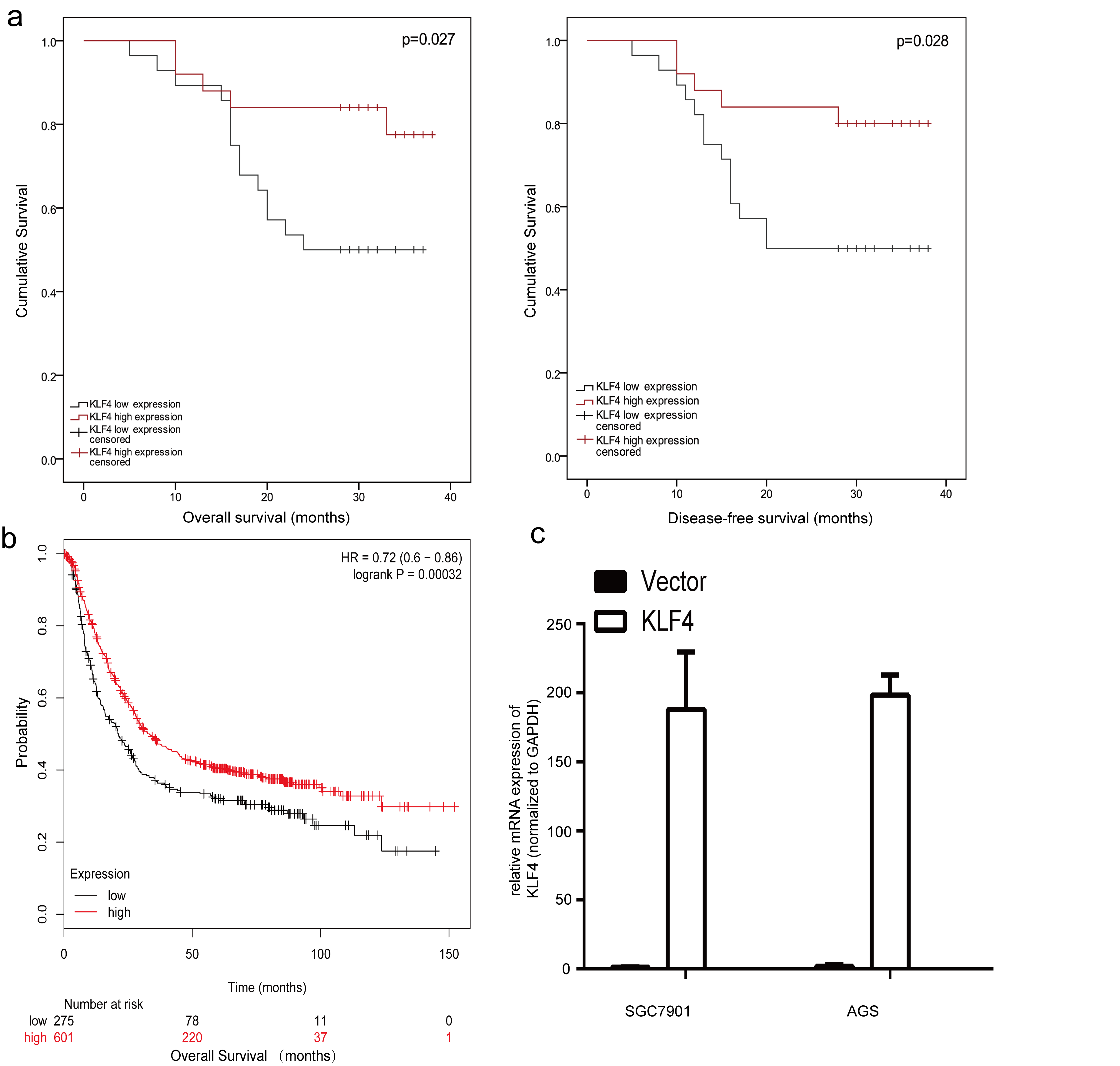


**S2. The correlation between KLF4 level and OS, DFS in GC tissues was determined by Kaplan-Meier method with log-rank test and Kaplan-Meier Plotter.** (**a**) The upper panels revealed that GC patients with high level of KLF4 had a better OS (p<0.001) and DFS (p<0.001). (**b**) the dataset from Kaplan-Meier Plotter indicated that GC patients with high level of KLF4 had a better OS (GSE14210, GSE15459, GSE22377, GSE29272, GSE51105, GSE62254)(p<0.05). (**c**) SGC7901 and AGS cell lines were transfected with KLF4 or control vector, and the mRNA level of KLF4 in SGC7901 and AGS cell lines were determined by qRT-PCR.


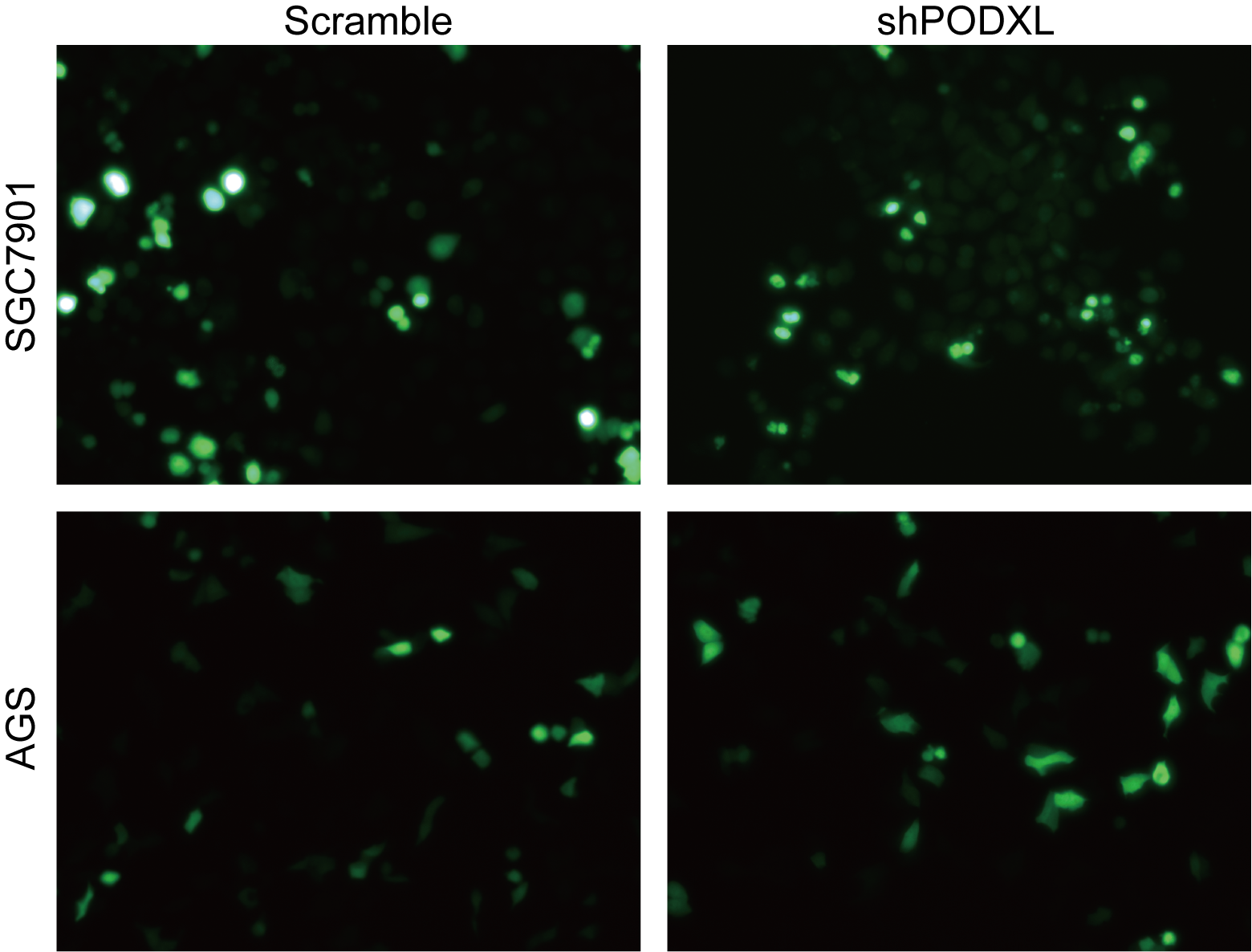


**S3. The level of PODXL in SGC7901 and AGS**. Representative images of stable cell lines of SGC7901 and AGS transfected with retrovirus vector or PODXL/shRNA.
